# Change in the prevalence of prehypertension and hypertension among young Indians aged 15–24 years between 2015–16 and 2019–21: Insights from nationally representative surveys

**DOI:** 10.1371/journal.pone.0319274

**Published:** 2025-04-01

**Authors:** Zahid Ali Khan, Uruj Qureshi, Tazeen Khan, Sonu Goel

**Affiliations:** 1 Department of Community Medicine, Government Medical College Baramulla, Jammu & Kashmir, India; 2 Department of Physiology, Government Medical College Srinagar, Jammu & Kashmir, India; 3 School of Medicine and Health Research Institute, University of Limerick, Limerick, Ireland; Bahir Dar University, ETHIOPIA

## Abstract

Globally, the prevalence of prehypertension and hypertension among adults in low- and middle-income countries is on the rise. However, the data on young people remains scarce. In this context, we analyzed data from two national-level cross-sectional surveys—NFHS–4, which included 272,966 individuals, and NFHS–5, which included 250,213 individuals—to assess changes in the prevalence of prehypertension and hypertension among young Indians aged 15–24 years. Between 2015–2016 and 2019–2021, the prevalence of prehypertension increased significantly (p <  0.001), rising from 38.9% to 44.5% among men and from 21.1% to 26.9% among women. While hypertension prevalence among men increased from 5.2% to 6.2%, it remained stable at approximately 4.0% among women over the same period. Most states, with a few exceptions, exhibited an increase in prehypertension prevalence across both genders, and more than two-thirds of states also showed an increase in hypertension prevalence among men. High BMI was found to be strongly associated with both prehypertension and hypertension. The rising prevalence of prehypertension and hypertension among young Indians aged 15–24 years is concerning and underscores the urgent need to develop targeted preventive strategies for this age group.

## Introduction

The United Nations defines youth as the transitional phase from childhood to adulthood, encompassing individuals aged 15 to 24 years [1]. This developmental period is marked by substantial changes across physical, psychological, neurocognitive, and social dimensions, significantly influencing overall health and well-being [2]. Despite the importance of this stage, young people are often marginalized in the context of universal health coverage, which is essential for meeting the Sustainable Development Goals [3]. This oversight stems from the assumption that this age group is inherently healthy, resulting in their exclusion from critical healthcare policy considerations. For instance, the National Non-Communicable Disease Control Programme of India currently recommends screening for non-communicable diseases only for individuals aged 30 years and above [4].

The global prevalence of prehypertension and hypertension is escalating, driven by population ageing and increased exposure to lifestyle risk factors such as poor dietary habits and insufficient physical activity [5]. Between 2000 and 2010, the prevalence of hypertension among individuals aged 20-29 years rose from 9.9% to 14.5% in men and from 5.4% to 9.4% in women. This increase was particularly notable in low- and middle-income countries, while high-income countries observed a decrease in hypertension prevalence over the same period [5]. In the United States, the prevalence of prehypertension among young adults aged 18 to 39 years decreased significantly from 32.2% in 1999 to 23.4% in 2014, although the prevalence of hypertension remained stable [6].

The data regarding trends in prehypertension and hypertension among individuals aged 15-24 years, especially in developing countries including India is lacking. A study conducted three decades ago among young Indians aged 15-24 years in New Delhi reported a hypertension prevalence of 3.1% (4.1% in men and 2.2% in women) [7]. More recent data from the National Capital Region, covering individuals aged 35–64 years between 1991–1994 and 2010–2012, revealed an increase in hypertension prevalence from 23.0% to 42.2% in urban areas and from 11.2% to 28.9% in rural areas [8]. However, there is a notable gap in recent data regarding the trend of prevalence of prehypertension and hypertension among young people in India over the past two decades.

Recent studies underscore the strong association between elevated blood pressure during youth and the risk of developing hypertension in later life, with significant implications for long-term cardiovascular health [9,10]. This highlights the critical need to explore the burden and risk factors associated with prehypertension and hypertension in young individuals. Accordingly, this study aims to leverage data from two national-level cross-sectional surveys conducted in 2015-16 (NFHS–4) and 2019-21 (NFHS–5) to assess changes in the prevalence of prehypertension and hypertension and to identify associated factors among young Indians aged 15-24 years.

## Materials and methods

### Study design

This study is a secondary data analysis comparing two cross-sectional surveys-the fourth round (NFHS–4) and the fifth round (NFHS–5) of the National Family Health Survey. The aim was to estimate changes in the prevalence of prehypertension and hypertension among men and women aged 15–24 years between these surveys. Data were collected nationwide, with NFHS–4 covering January 2015 to December 2016 and NFHS–5 covering June 2019 to April 2021.

### Study population

**Fig 1:**
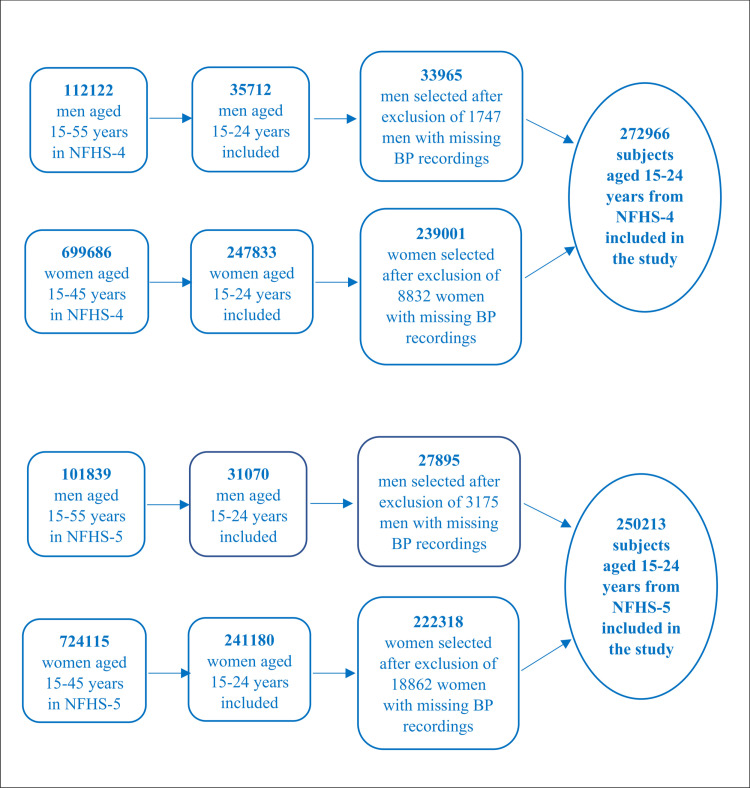
Flow chart depicting the selection of study subjects from the two survey cycles after various exclusions.

The study included men and women aged 15–24 years from all districts across India, using data from NFHS–4 (2015–16) and NFHS–5 (2019–21). The analysis involved 272,966 individuals from NFHS–4 and 250,213 individuals from NFHS–5 (Fig 1).

### Sampling strategy

A uniform sample design, representative at the national, state/union territory, and district levels, was employed in each survey round. Both surveys used a two-stage stratified random sampling approach, with the 2011 census serving as the sampling frame. In the first stage, villages in rural areas and Census Enumeration Blocks (CEBs) in urban areas were selected as Primary Sampling Units (PSUs) through the Probability Proportional to Size (PPS) method. In the second stage, a complete household listing was conducted in each selected rural and urban PSU, followed by the random selection of 22 households using a systematic sampling technique.

Stratification was done in both rural and urban areas which was crucial for capturing sociodemographic diversity. Rural PSUs were stratified by village population, the proportion of Scheduled Castes (SCs) and Scheduled Tribes (STs), and further sorted by women’s literacy rates. In urban areas, PSUs were stratified based on SC/ST population proportions.

### Data collection and processing

The surveys ensured high-quality data collection by employing a rigorous sampling design and precise data collection methods. Throughout the two surveys, electronic data was collected daily using the SyncCloud system at the International Institute for Population Sciences (IIPS) and stored securely. Enumerators underwent thorough training, and pre-testing of questionnaires ensured clarity and cultural appropriateness. Quality control measures were implemented during fieldwork, standardized procedures were followed, and data verification and cleaning processes were employed post-collection; all contributing to the reliability and accuracy of the survey data.

Secondary editing addressed computer-identified inconsistencies and coded open-ended responses, conducted both in the field and at the central office, with the International Institute for Population Sciences (IIPS) verifying the edits before finalizing the dataset. Regular field-check tables were generated to identify errors in data collection, and timely feedback regarding the performance of fieldwork teams and individual investigators was provided to facilitate necessary improvements.

Data for both surveys were collected using four questionnaires—‘household,’ ‘women,’ ‘men,’ and ‘biomarker’—administered in 18 local languages via Computer Assisted Personal Interviewing (CAPI). CAPI ensures data quality and comparability at district, state and national levels by automatically following skip patterns and filters. Interviewers made at least three visits to households if eligible members were not available during initial visits.

Households were categorized into wealth quintiles—‘poorest,’ ‘poorer,’ ‘middle,’ ‘richer,’ and ‘richest’—based on living standards, including housing conditions, assets, and access to essential services [11]. Weight and height were measured using a Seca 874 digital scale and Seca 213 stadiometer, respectively, to calculate body mass index (BMI).

Blood pressure was recorded through a standardized protocol using an Omron Automatic digital blood pressure monitor. Participants were seated comfortably for at least five minutes prior to measurement. Blood pressure was measured on the upper arm, utilizing appropriately sized cuffs based on the participant’s arm circumference. Three readings were taken at five-minute intervals, with the average of the last two readings used for analysis. In cases where only two blood pressure recordings were available, the average of those two readings was calculated. If only one recording was available, that reading was considered the final measurement.

Prehypertension was defined according to the Seventh Report of the Joint National Committee (JNC-7) as systolic blood pressure (SBP) of 120–139 mmHg and/or diastolic blood pressure (DBP) of 80–89 mmHg. Hypertension was defined as SBP ≥ 140 mmHg and/or DBP ≥ 90 mmHg, or if the individual was on antihypertensive medication [12].

BMI was calculated as weight divided by height squared (kg/m²) and categorized into four groups according to BMI classification for Asian Indians [13]: ‘Underweight’ (BMI < 18.0 kg/m²), ‘Normal’ (BMI 18.0–22.9 kg/m²), ‘Overweight’ (BMI 23.0–24.9 kg/m²), and ‘Obese’ (BMI ≥ 25.0 kg/m²).

### Dependent variables

Two dependent variables taken for this study were Prehypertension and Hypertension.

Prehypertension is a category between normotension and hypertension as proposed by the Seventh Report of the Joint National Committee (JNC-7). It was classified into a dichotomous variable – prehypertensive and normotensive.

Hypertension was classified into a dichotomous variable – hypertensive and non-hypertensive, based on JNC 7 classification [12] and a history of antihypertensive medication intake.

### Independent variables

Independent variables included sociodemographic factors, BMI, tobacco use, alcohol consumption, and dietary habits.

### Statistical analysis

Data from eligible young adults were analyzed using SPSS version 20. Subjects with missing blood pressure recordings were excluded from the analysis. Sample weights provided in the datasets were employed to adjust the survey data, ensuring it was more representative of the studied population and minimizing potential bias in the two survey samples. Percentage changes in the prevalence of prehypertension and hypertension between NFHS–4 and NFHS–5 were calculated. Chi-square tests assessed associations between prehypertension, hypertension, and various factors. Confounding factors were controlled using binary logistic regression analysis with the ‘Enter’ method, applied to variables that exhibited significant associations (p <  0.05) in the univariate analysis. Adjusted odds ratios with 95% confidence intervals were calculated, and p-values < 0.05 were considered statistically significant.

### Ethical clearance

The NFHS adheres to stringent ethical protocols throughout data collection and processing, including informed consent, risk and benefit assessment, voluntary participation, and ensuring privacy and confidentiality. Gender-sensitive team composition, with a mix of men and women investigators proficient in local languages, is maintained. The study received approval from the Institutional Ethics Committee of the Postgraduate Institute of Medical Education and Research, Chandigarh, India (vide no. PGI/IEC/2022/001090 dated 09/09/2022).

## Results

In NFHS-4 (2015-16), of the 272,966 individuals aged 15-24 years, 33,965 (14.2%) were men and 239,001 (85.8%) were women. In NFHS-5 (2019-21), out of 250,213 individuals, 27,895 (12.5%) were men and 222,318 (87.5%) were women. The distribution of sociodemographic characteristics remained similar across the two surveys. However, the proportion of married individuals decreased from 12.7% to 10.9% among men and from 38.3% to 34.5% among women. Literacy improved, with illiteracy among women decreasing from 10.8% to 6.7% and among men from 5.3% to 4.6%.

Obesity increased from 7.1% to 9.1% among men and from 6.7% to 8.3% among women. Behavioural factors improved, with reductions in tobacco smoking (15.3% to 11.4% among men, 0.3% to 0.2% among women), smokeless tobacco use (22.4% to 17.8% among men, 4.0% to 2.3% among women), and alcohol consumption (17.8% to 12.3% among men, 1.3% to 0.7% among women) (Table 1).

**Table 1:** Background characteristics of young Indians (15-24 years) in 2015-16 and 2019-21.

Characteristics	Men				Women			
	2015-16		2019-21		2015-16		2019-21	
**Place of residence**								
*Urban*	10489	30.9%	6869	24.6%	65354	27.3%	49207	22.1%
*Rural*	23476	69.1%	21026	75.4%	173647	72.7%	173111	77.9%
**Ethnicity**								
*Scheduled Caste*	6449	20.0%	5544	20.7%	44880	19.6%	45819	21.6%
*Scheduled Tribe*	5744	17.8%	5238	19.5%	42057	18.3%	41468	19.5%
*Others*	20122	62.3%	16027	59.8%	142422	62.1%	125225	58.9%
**Religion**								
*Hindu*	25244	74.3%	21467	77.0%	176515	73.9%	169338	76.2%
*Muslim*	5150	15.2%	3227	11.6%	36744	15.4%	28962	13.0%
*Christian*	2075	6.1%	1873	6.7%	15775	6.6%	14627	6.6%
*Sikh*	749	2.2%	657	2.4%	4644	1.9%	4161	1.9%
*Others*	747	2.2%	671	2.4%	5323	2.2%	5230	2.4%
**Marital status**								
*Never married*	29584	87.1%	24800	88.9%	145915	61.1%	144219	64.9%
*Married*	4310	12.7%	3040	10.9%	91455	38.3%	76789	34.5%
*Widowed/Divorced/Separated*	71	0.2%	55	0.2%	1631	0.7%	1310	0.6%
**Educational level**								
*No education*	1811	5.3%	1281	4.6%	25762	10.8%	14917	6.7%
*Primary*	2565	7.6%	1541	5.5%	21591	9.0%	14005	6.3%
*Secondary*	24219	71.3%	20054	71.9%	158020	66.1%	154947	69.7%
*Higher*	5370	15.8%	5019	18.0%	33628	14.1%	38449	17.3%
**Wealth index**								
*Poorest*	5704	16.8%	5692	20.4%	46540	19.5%	49334	22.2%
*Poorer*	7543	22.2%	6632	23.8%	54735	22.9%	53057	23.9%
*Middle*	7663	22.6%	5981	21.4%	52459	21.9%	47455	21.3%
*Richer*	6869	20.2%	5234	18.8%	46272	19.4%	41110	18.5%
*Richest*	6186	18.2%	4356	15.6%	38995	16.3%	31362	14.1%
**Body mass index**								
*Underweight*	8699	25.6%	6204	22.3%	61321	25.7%	53112	23.9%
*Normal*	19926	58.7%	16223	58.3%	143052	59.9%	131270	59.0%
*Overweight*	2943	8.7%	2851	10.2%	18662	7.8%	19474	8.8%
*Obese*	2397	7.1%	2543	9.1%	15966	6.7%	18459	8.3%
**Tobacco smoking**								
*No*	28766	84.7%	24714	88.6%	238224	99.7%	221867	99.8%
*Yes*	5199	15.3%	3181	11.4%	777	0.3%	451	0.2%
**Smokeless Tobacco use**								
*No*	26361	77.6%	22935	82.2%	229376	96.0%	217157	97.7%
*Yes*	7604	22.4%	4960	17.8%	9625	4.0%	5161	2.3%
**Alcohol intake**								
*No*	27913	82.2%	24474	87.7%	235882	98.7%	220686	99.3%
*Yes*	6052	17.8%	3421	12.3%	3119	1.3%	1632	0.7%
**Dietary habits**								
*Vegetarian*	10909	32.1%	6117	21.9%	95493	40.0%	67129	30.2%
*Nonvegetarian*	23056	67.9%	21778	78.1%	143508	60.0%	155189	69.8%

### Prevalence

#### Prehypertension.

The prevalence of prehypertension increased significantly (p < 0.001) from 38.9% (95% CI; 38.4-39.5) in 2015-16 to 44.5% (95% CI;43.9-45.1) in 2019-21 among men and from 21.1% (95% CI;20.9-21.3) in 2015-16 to 26.9% (95% CI;26.7-27.1) in 2019-21 among women. (Table 2)

**Table 2:** Comparison of prehypertension and hypertension among study participants between 2015-16 and 2019-21.

		Prevalence % (95% CI)		
		2015-16	2019-21	p value
Prehypertension	Men	38.9 (38.4 - 39.5)	44.5 (43.9 - 45.1)	<0.001
	Women	21.1 (20.9 - 21.3)	26.9 (26.7 - 27.1)	<0.001
Hypertension	Men	5.2 (5.0 - 5.4)	6.2 (5.9 - 6.5)	<0.001
	Women	4.1 (4.0 - 4.1)	4.0 (3.9 - 4.1)	0.194

**Fig 2:**
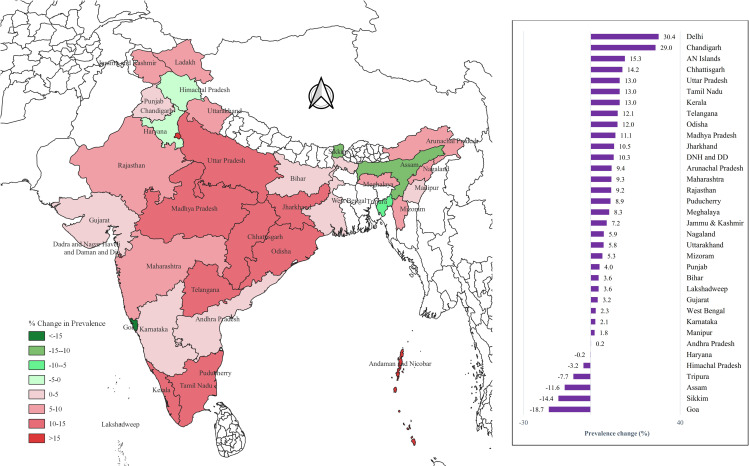
State-wise change in the prevalence of prehypertension among men aged 15-24 years. (Map prepared in QGIS version 3.20.0).

**Fig 3:**
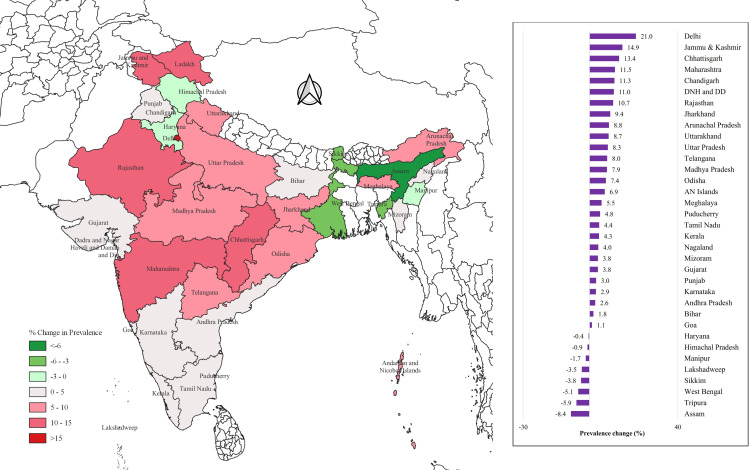
State-wise change in the prevalence of prehypertension among women aged 15-24 years. (Map prepared in QGIS version 3.20.0).

Between 2015–2016 and 2019–2021, most states and Union Territories (UTs) in India showed an increase in the prevalence of prehypertension among both men and women. The highest increase was reported in Delhi NCR, with a rise of 30.4% in men and 21% in women. In contrast, the largest decline in prehypertension prevalence was observed in Goa among men (18.7%) and in Assam among women (8.4%) (Fig 2–3).

#### Hypertension.

The prevalence of hypertension among men exhibited a statistically significant increase, rising from 5.2% (95% CI: 5.0–5.4) in 2015–2016 to 6.2% (95% CI: 5.9–6.5) in 2019–2021 (p <  0.001). In contrast, no notable change was observed in the prevalence of hypertension among women during the same period (Table 2).

**Fig 4:**
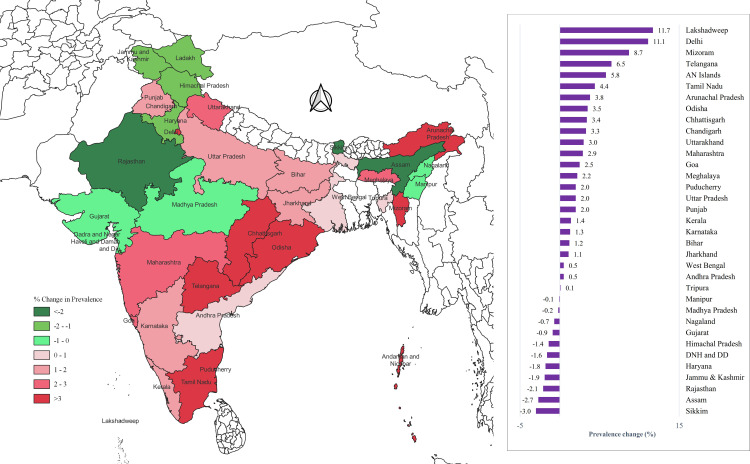
State-wise change in the prevalence of hypertension among men aged 15-24 years. (Map prepared in QGIS version 3.20.0).

**Fig 5:**
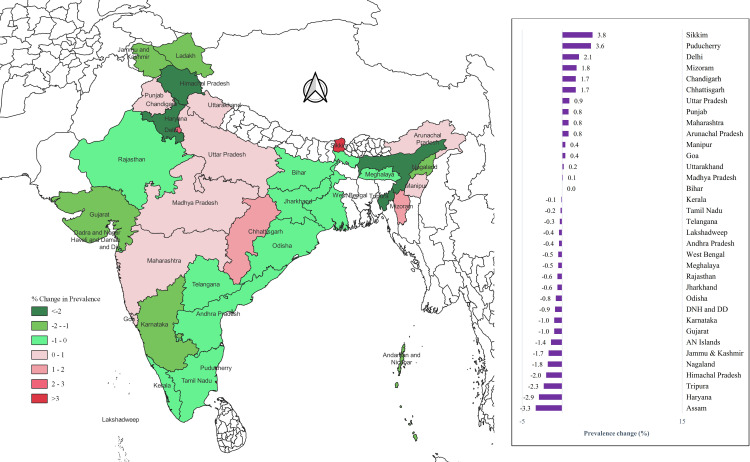
State-wise change in the prevalence of hypertension among women aged 15-24 years. (Map prepared in QGIS version 3.20.0).

Although the overall hypertension prevalence among women remained stable between 2015–2016 and 2019–2021, most states and Union Territories (UTs) experienced a decline. The most pronounced increases were recorded in Lakshadweep, with an 11.7% rise among men, and in Sikkim, with a 3.8% rise among women. Conversely, Sikkim saw the largest decline in hypertension prevalence (3%) among men, while Assam exhibited the most significant reduction among women (3.3%) (Fig 4–5).

### Associated factors

#### Prehypertension.

Logistic regression analysis indicated that women subjects residing in rural areas had significantly higher odds of prehypertension compared to those living in urban areas across both surveys. In contrast, no such association was found among men. Additionally, individuals belonging to Scheduled Tribes, those practising the Sikh religion, and those who were uneducated or married exhibited markedly higher odds of prehypertension across both genders. Among women, those classified in the poorest wealth index category showed the highest likelihood of prehypertension compared to other wealth categories (Table 3).

**Table 3:** Factors associated with prehypertension among study participants in the year 2015-16 and 2019-21.

	Men AOR (95% CI)		Women AOR (95% CI)	
	2015-16	2019-21	2015-16	2019-21
**Place of residence**				
*Urban*		1	1	1
*Rural*	–	0.99 (0.93-1.06)	1.31 (1.27-1.34)^*^	1.07 (1.05-1.10)^*^
**Ethnicity**				
*Others*	1	1	1	1
*Scheduled Caste*	1.01 (0.95-1.07)	1.05 (0.99-1.13)	1.02 (0.99-1.05)	1.03 (1.01-1.06)^*^
*Scheduled Tribe*	1.33 (1.23-1.45)^*^	1.34 (1.22-1.47)^*^	1.12 (1.08-1.17)^*^	1.35 (1.30-1.39)^*^
**Religion**				
*Hindu*	1	1	1	1
*Muslim*	0.92 (0.85-0.99)^*^	0.98 (0.91-1.06)	1.19 (1.15-1.22)^*^	1.21 (1.18-1.25)^*^
*Christian*	0.79 (0.67-0.94)^*^	0.86 (0.73-1.02)	0.91 (0.84-0.98)^*^	0.80 (0.74-0.86)^*^
*Sikh*	1.77 (1.47-2.12)^*^	1.56 (1.21-2.02)^*^	1.75 (1.62-1.89)^*^	1.22 (1.13-1.33)^*^
*Others*	1.05 (0.86-1.28)	0.93 (0.75-1.16)	1.01 (0.93-1.10)	1.17 (1.06-1.28)^*^
**Marital status**				
*Never married*	1	1	1	1
*Married*	1.16 (1.08-1.24)^*^	1.19 (1.09-1.30)^*^	1.06 (1.04-1.09)^*^	1.00 (0.98-1.02)
*Widowed/Divorced/Separated*	1.31 (0.75-2.28)	0.92 (0.52-1.62)	0.88 (0.78-1.01)	0.79 (0.68-0.91)^*^
**Educational level**				
*No education*	1	1	1	1
*Primary*	0.98 (0.85-1.11)	0.65 (0.55-0.77)^*^	0.98 (0.94-1.02)	0.98 (0.93-1.03)
*Secondary*	0.86 (0.77-0.96)^*^	0.78 (0.69-0.89)^*^	0.80 (0.77-0.83)^*^	0.78 (0.75-0.81)^*^
*Higher*	1.03 (0.91-1.17)	0.97 (0.85-1.12)	0.88 (0.84-0.92)^*^	0.83 (0.79-0.87)^*^
**Wealth index**				
*Poorest*	1	1	1	1
*Poorer*	1.05 (0.97-1.13)	1.10 (1.02-1.20)^*^	0.94 (0.91-0.97)^*^	0.93 (0.90-0.96)^*^
*Middle*	1.03 (0.95-1.12)	1.12 (1.02-1.22)^*^	0.84 (0.81-0.87)^*^	0.95 (0.92-0.98)^*^
*Richer*	0.93 (0.85-1.01)	1.01 (0.92-1.11)	0.80 (0.77-0.83)^*^	0.96 (0.93-0.99)^*^
*Richest*	1.04 (0.95-1.13)	0.97 (0.87-1.08)	0.85 (0.82-0.89)^*^	0.99 (0.95-1.03)
**Body mass index**				
*Underweight*	1	1	1	1
*Normal*	2.27 (2.14-2.40)^*^	1.92 (1.80-2.05)^*^	1.42 (1.38-1.46)^*^	1.41 (1.37-1.44)^*^
*Overweight*	3.93 (3.58-4.30)^*^	3.20 (2.90-3.54)^*^	2.16 (2.07-2.24)^*^	1.89 (1.82-1.96)^*^
*Obese*	4.58 (4.16-5.04)^*^	4.26 (3.85-4.71)^*^	3.06 (2.94-3.18)^*^	2.46 (2.38-2.56)^*^
**Tobacco smoking**				
*No*	1	1		
*Yes*	1.10 (1.02-1.19)^*^	0.99 (0.91-1.09)	–	–
**Smokeless Tobacco use**				
*No*	1	1	1	
*Yes*	1.13 (1.06-1.20)^*^	1.02 (0.94-1.10)	1.11 (1.03-1.19)^*^	–
**Alcohol intake**				
*No*	1	1	1	
*Yes*	1.06 (0.99-1.14)	1.15 (1.05-1.26)^*^	1.17 (1.04-1.33)^*^	–
**Dietary habits**				
*Vegetarian*	1			1
*Nonvegetarian*	0.89 (0.85-1.04)	–	–	0.78 (0.76-1.08)

The likelihood of prehypertension was significantly associated with increasing BMI for both men and women during 2015–2016 and 2019–2021. Among men, tobacco smoking and smokeless tobacco use were linked to higher odds of prehypertension in 2015–2016, while alcohol consumption was associated with significantly increased odds in 2019–2021. For women, significant associations with prehypertension were observed with smokeless tobacco use and alcohol intake during 2015–2016. Additionally, no significant correlation was found between dietary habits and prehypertension in either gender (Table 3).

#### Hypertension.

Logistic regression analysis uncovered significant variations in hypertension odds based on geographic and demographic factors. Men residing in rural areas exhibited significantly lower odds of hypertension during 2019–2021, whereas women from rural areas demonstrated higher odds in 2015–2016. Both genders showed elevated odds of hypertension if they were Sikh by religion or married. Among women, those from Scheduled Tribes, without formal education, and in the poorest wealth index category had substantially higher odds of hypertension (Table 4).

**Table 4:** Factors associated with hypertension among study participants in the year 2015-16 and 2019-21.

	Men AOR (95% CI)		Women AOR (95% CI)	
	2015-16	2019-21	2015-16	2019-21
**Place of residence**				
*Urban*		1	1	
*Rural*	–	0.78 (0.69-0.88)^*^	1.16 (1.10-1.22)^*^	–
**Ethnicity**				
*Others*			1	1
*Scheduled Caste*	–	–	0.97 (0.91-1.02)	0.97 (0.92-1.02)
*Scheduled Tribe*	–	–	1.07 (0.99-1.15)	1.16 (1.08-1.25)^*^
**Religion**				
*Hindu*	1	1	1	1
*Muslim*	0.90 (0.77-1.04)	0.73 (0.62-0.85)^*^	1.19 (1.12-1.27)^*^	1.05 (0.98-1.13)
*Christian*	1.10 (0.79-1.53)	0.85 (0.61-1.19)	1.03 (0.88-1.20)	0.99 (0.85-1.15)
*Sikh*	1.80 (1.33-2.42)^*^	1.78 (1.18-2.69)^*^	1.42 (1.22-1.66)^*^	1.71 (1.46-2.01)^*^
*Others*	1.47 (1.02-2.11)^*^	0.64 (0.40-1.02)	0.95 (0.80-1.13)	1.03 (0.83-1.28)
**Marital status**				
*Never married*	1	1	1	1
*Married*	1.33 (1.16-1.53)^*^	1.48 (1.28-1.71)^*^	1.26 (1.21-1.32)^*^	1.18 (1.13-1.24)^*^
*Widowed/Divorced/ Separated*	2.20 (0.88-5.49)	0.97 (0.34-2.79)	1.37 (1.08-1.74)^*^	1.02 (0.75-1.37)
**Educational level**				
*No education*	1	1	1	1
*Primary*	1.11 (0.84-1.46)	0.60 (0.44-0.81)^*^	0.97 (0.89-1.06)	0.94 (0.84-1.05)
*Secondary*	0.89 (0.71-1.12)	0.72 (0.57-0.90)^*^	0.90 (0.84-0.97)^*^	0.82 (0.76-0.90)^*^
*Higher*	1.23 (0.96-1.58)	0.67 (0.52-0.87)^*^	1.00 (0.91-1.09)	0.92 (0.83-1.01)
**Wealth index**				
*Poorest*	1	1	1	1
*Poorer*	0.98 (0.82-1.17)	1.13 (0.95-1.34)	0.96 (0.90-1.03)	0.95 (0.89-1.02)
*Middle*	1.04 (0.87-1.24)	1.51 (0.91-1.78)	0.86 (0.80-0.92)^*^	0.91 (0.85-0.98)^*^
*Richer*	1.05 (0.88-1.26)	0.84 (0.69-1.02)	0.97 (0.90-1.05)	0.86 (0.80-0.93)^*^
*Richest*	1.06 (0.88-1.28)	0.97 (0.79-1.20)	0.97 (0.89-1.06)	0.80 (0.74-0.87)^*^
**Body mass index**				
*Underweight*	1	1	1	1
*Normal*	1.77 (1.53-2.05)^*^	1.66 (1.43-1.93)^*^	1.14 (1.08-1.20)^*^	1.09 (1.03-1.16)^*^
*Overweight*	2.90 (2.40-3.51)^*^	2.41 (1.98-2.93)^*^	1.40 (1.28-1.52)^*^	1.54 (1.41-1.67)^*^
*Obese*	4.86 (4.07-5.81)^*^	4.50 (3.77-5.37)^*^	2.36 (2.19-2.54)^*^	2.24 (2.07-2.41)^*^
**Tobacco smoking**				
*No*				
*Yes*	1.01 (0.86-1.17)	–	–	–
**Smokeless Tobacco use**				
*No*			1	1
*Yes*	1.16 (1.01-1.32)^*^	–	1.10 (0.96-1.26)	1.16 (0.97-1.39)
**Alcohol intake**				
*No*		1		
*Yes*	1.16 (1.01-1.33)^*^	1.28 (1.11-1.49)^*^	1.33 (1.05-1.67)^*^	–
**Dietary habits**				
*Vegetarian*				
*Nonvegetarian*	–	–	1.00 (0.95-1.04)	–

Furthermore, a notable direct relationship was observed in increasing BMI with increasing odds of hypertension for both genders. Smokeless tobacco use was associated with higher odds of hypertension, though this association was not statistically significant among women. Alcohol consumption, however, was linked to significantly higher odds of hypertension for both men and women. In contrast, tobacco smoking and dietary habits did not show a significant association with hypertension (Table 4).

## Discussion

The representative samples from India have demonstrated a marked increase in the prevalence of prehypertension among young men and women aged 15-24 years between 2015-16 and 2019-21. During this period, hypertension prevalence escalated solely among men, while remaining static for women. Furthermore, there was a discernible reduction in the proportion of married individuals and a significant improvement in literacy rates. These trends are interrelated, as women with lower educational attainment tend to marry at an earlier age compared to those with higher education [14,15].

Additionally, both surveys revealed a decline in the prevalence of tobacco use and alcohol consumption, mirroring a global trend and likely attributable to heightened awareness and extensive tobacco control initiatives [16,17]. Furthermore, there was a rise in the prevalence of overweight and obesity among both genders from 2015-16 to 2019-21, reflecting global patterns of rising average body mass index (BMI) values [18,19].

### Prehypertension

In our study, we observed around 5.6% rise in the prevalence of prehypertension among young men, increasing from 38.9% in 2015-16 to 44.5% in 2019-21. Similarly, among young women, the prevalence grew by 5.8% from 21.1% to 26.9% over the same period. The highest increase in prehypertension prevalence among men was observed in the northern region of Delhi NCR, while the greatest decline was observed in Goa. Among women, Delhi NCR also reported the highest increase in prehypertension prevalence, whereas Assam in the northeastern region showed the most substantial decline. These regional disparities in prevalence seem to be influenced by a complex interplay of social, cultural, dietary, and physical activity factors, as highlighted by a 2011 study conducted across five Indian states, which reported the highest prevalence in South India [20].

Data on the trend of prehypertension among young people aged 15-24 years is limited, with no existing literature from India. However, a few studies from developing countries have reported an increase in the prevalence of prehypertension, which aligns with our findings [21,22]. A study of Iranian youth aged 15-19 years revealed a rise in prehypertension prevalence for both genders between 2007 and 2011 [21]. Similarly, Taiwanese adolescents experienced a significant increase in prehypertension prevalence among boys from 1996 to 2006, though no change was noted among girls [22]. In contrast, developed countries are reporting a decline in prehypertension rates. For instance, a study from the United States indicated an 8.8% reduction in prehypertension prevalence among young adults aged 18–39 years between 1999 and 2014 [6]. This decline in the United States has been attributed to reductions in smoking rates and dietary improvements among the youth [6].

In the current study, the burden of prehypertension in young people (15-24 years) during 2019-21 was estimated at 44.5% among men, and 26.9% among women. Almost similar findings on the prevalence of prehypertension among young people have been reported in some studies from India. A prior study by Gupta R et al., conducted across 11 cities in Northern, Southern, Eastern, and Western Indian states, reported prehypertension rates of 43.7% in men and 33.3% in women aged 20-29 years in 2012 [23]. Another study conducted among young adults aged 20–39 years in South India reported a prehypertension prevalence of 47.5% in men and 26.1% in women, which is comparable to our findings [24]. Additionally, a separate study from South India found a prehypertension prevalence of 33.3% among young people aged 18-19 years [25]. Likewise, a significant burden of prehypertension among young adults has been highlighted in recent studies from various countries, including Iran [21], Tanzania and Uganda [26], Peru [27], China [28], Korea [29], Turkey [30] and Dubai [31]. The increasing prevalence of prehypertension poses a serious concern, as it is closely linked to subclinical atherosclerosis and damage to target organs, potentially leading to substantial increases in morbidity and mortality in later life [32].

### Hypertension

The prevalence of hypertension among men escalated from 5.2% in 2015-16, to 6.2% in 2019-21, while the prevalence among women remained relatively stable at 4.1% in 2015-16 and 4.0% in 2019-21. Lakshadweep and Sikkim reported the highest increases in hypertension prevalence among men and women, respectively, while Sikkim and Assam saw the most significant decreases in prevalence between 2014-15 and 2019-21. These regional disparities in prevalence seem to be influenced by a complex interplay of social, cultural, dietary, and physical activity factors, as highlighted by a 2011 study conducted across five Indian states, which reported the highest prevalence in South India [20].

The literature on the trend of hypertension among young people in India is not available. However, the trend observed in our study mirrors findings from Germany, where a comparison of cross-sectional surveys conducted in 1998 and 2008-11 revealed an increase in hypertension prevalence among men aged 18-29 years, with no change observed among women [33]. In contrast, a significant increase in hypertension prevalence was documented among both men and women adolescents in Taiwan from 1996 to 2006, as well as among Iranian adolescents aged 15-19 years between 2007 and 2011 [21,22]. African nations, such as Nigeria, reported a dramatic increase in hypertension prevalence, rising from 1.3% to 29.6% in men and from 0.6% to 17.1% in women aged 20-24 years between 1995 and 2020 [34]. Conversely, in developed countries like the United States, the prevalence of hypertension among young adults aged 18–39 years remained unchanged from 1999 to 2014 [6].

In the current study, the prevalence of hypertension during 2019-2021 was estimated to be approximately 6.2% in men and 4.0% in women. This finding aligns with a systematic review and meta-analysis of cross-sectional studies conducted among adolescents (ages 10-19 years) in India, which reported hypertension prevalence ranging from 2% to 20.5%, with a pooled estimate of 7.6% [35]. Most studies included in the review reported hypertension prevalence below 10%, with Gupta et al [23] documenting the highest prevalence at 20.5%, likely due to the use of a modified ATP classification for diagnosing hypertension which could have overestimated the prevalence in their analysis.

Another systematic review and meta-analysis conducted by de Moraes [36] reported a hypertension prevalence of 11.2% among adolescents from both developed and developing countries, which is higher than the prevalence found in our study. This discrepancy may be attributed to the inclusion of studies utilizing the modified ATP classification for elevated blood pressure.

Evidence from NHANES 2017-18 survey reveals an estimated hypertension prevalence of 7% among US young adults aged 18 to 39 years. A study from China reported a much lower prevalence (1.9%) among individuals aged 15-24 years compared to our study [28]. Conversely, higher rates of hypertension (10%) have been documented in studies from Tanzania and Uganda among individuals aged 20-25 years [26]. Notably, an exceptionally high burden of hypertension has been reported among young adults aged 18-29 years in Dubai (19.89%) [31], and among those aged 20-29 years in Turkey (19.1%) [30].

### Associated factors

Multiple factors contribute to elevated blood pressure among young adults. Sociodemographic variables and body mass index demonstrated a consistent association with prehypertension in both men and women during 2015-16 and 2019-21. However, its association with tobacco use and alcohol consumption varied between the two survey periods. Consistently associated factors with hypertension among both genders included religion, marital status and body mass index, however, alcohol intake depicted an association among men only.

In 2019-21, the prevalence of prehypertension and hypertension was higher in urban areas among men. Conversely, women in rural areas exhibited a greater prevalence of both conditions compared to their urban counterparts, although this rural-urban disparity in prevalence lessened by 2019-21. A study involving Chinese children and adolescents aged 7 to 18 years also reported a higher prevalence of hypertension in rural areas, with a decreasing trend in urban-rural disparity from 1995 to 2014 [37]. Similar rural-urban disparities in hypertension prevalence have been documented in studies from various countries [38,39]. The increasing prevalence of high blood pressure in urban areas, likely driven by environmental factors and the adoption of unhealthy lifestyles resulting from rapid economic development, may account for both the rising prevalence and the reduction in the urban-rural disparity [40].

Evidence from NHANES and other studies underscores the rising prevalence of childhood obesity as a significant determinant of the increasing rates of hypertension among young individuals [41,42]. This surge in overweight and obesity among the youth is likely linked to modernization and lifestyle changes associated with evolving socio-economic conditions [43]. A study among Taiwanese adolescents corroborated this trend, revealing an increase in prehypertension and hypertension, particularly among those who are overweight [22]. However, it is noteworthy that our observations indicate a decrease in the strength of the association between high BMI and hypertension from 2015-16 to 2019-21, as evidenced by a reduction in adjusted odds ratios. This suggests the growing influence of other risk factors on prehypertension and hypertension, warranting further investigation to enhance blood pressure management among young people.

This study boasts several strengths, including a large sample size that provides robust estimates and allows for the assessment of the burden of prehypertension and hypertension at the state level also, facilitating reliable local and international comparisons. However, the study also has some limitations. Notably, there is a skewed representation of the population toward women, and the cross-sectional design of the study can only establish associations, not causality.

## Conclusion

In summary, the data indicates a significant rise in the prevalence of prehypertension among young men and women aged 15-24 years in India between 2015-16 and 2019-21. Notably, the increase in hypertension prevalence was observed solely in men. The burden of prehypertension and hypertension in India remains alarmingly high, comparable to findings from numerous studies worldwide. While some countries have reported declines in prevalence, these changes may stem from complex interactions between lifestyle, socio-economic factors, and public health interventions. Additionally, we observed a decrease in tobacco and alcohol use, reflecting global trends. The rising prevalence of prehypertension and hypertension among young people aged 15-24 years underscores the urgent need for effective management and preventive strategies targeted at this age group.

## Recommendations

Given that nearly half of the young men and more than one-fourth of the young women in India are currently prehypertensive, with a significant risk of progressing to hypertension and related complications, there is an urgent need to develop targeted preventive strategies for this age group. Current healthcare policies on non-communicable disease prevention and control primarily focus on individuals aged 30 years and above, as outlined in national programs. Therefore, it is essential to reduce the screening age for high blood pressure by at least a decade. Such a change could substantially reduce the overall burden of hypertension in India and mitigate associated morbidity and mortality.
